# Young parents produce offspring with short telomeres: A study in a long-lived bird, the Black-browed Albatross (*Thalassarche melanophrys*)

**DOI:** 10.1371/journal.pone.0193526

**Published:** 2018-03-21

**Authors:** Sophie Marie Dupont, Christophe Barbraud, Olivier Chastel, Karine Delord, Stéphanie Ruault, Henri Weimerskirch, Frédéric Angelier

**Affiliations:** Centre d’Etudes Biologiques de Chizé, CNRS, ULR, Villiers en Bois, France; Phillip Island Nature Parks, AUSTRALIA

## Abstract

In wild vertebrates, young parents are less likely to successfully rear offspring relative to older ones because of lower parental skills (‘the constraint hypothesis’), lower parental investment (‘the restraint hypothesis’) or because of a progressive disappearance of lower-quality individuals at young ages (‘the selection hypothesis’). Because it is practically difficult to follow an offspring during its entire life, most studies have only focused on the ability of individuals to breed or produce young, while neglecting the ability of such young to subsequently survive and reproduce. Several proxies of individual quality can be useful to assess the ability of young to survive and recruit into the population. Among them, telomere length measurement appears especially promising because telomere length has been linked to longevity and fitness in captive and wild animals. By sampling 51 chicks reared by known-aged parents, we specifically tested whether parental age was correlated to offspring telomere length and body condition in a long-lived bird species, the Black-browed Albatross (*Thalassarche melanophrys*). Young Black-browed albatrosses produced chicks with shorter telomere relative to those raised by older ones. Short offspring telomeres could result from poor developmental conditions or heritability of telomere length. Moreover, young parents also had chicks of lower body condition when compared with older parents, although this effect was significant in female offspring only. Overall, our study demonstrates that parental age is correlated to two proxies of offspring fitness (body condition and telomere length), suggesting therefore that older individuals provide better parental cares to their offspring because of increased parental investment (restraint hypothesis), better foraging/parental skills (constraint hypothesis) or because only high-quality individuals reach older ages (selection hypothesis).

## Introduction

In wild vertebrates, age has a strong influence on an individual’s reproductive performances, which typically increase with age before stabilizing at middle age and declining in senescent individuals [1]. In particular, young individuals have poorer breeding success relative to middle-aged individuals. Several hypotheses have been proposed to explain the poorer performances of young individuals [1,2,3]. The improvement of performances with advancing age could result from selective processes and progressive disappearance of low quality parents (“the selection hypothesis”) and/or from an increased parental investment from older individuals (“the restraint hypothesis”). Furthermore, “the constraint hypothesis” proposes that young individuals have not yet acquired or developed the skills that are required to breed and to cope with environmental constraints [3]. For example, young individuals are usually more likely to be predated, have a lower foraging efficiency [4,5], a lower social rank [6,7], and therefore, have overall a lower survival probability than older individuals [8,9,10]. Several studies have also reported that young breeders are less likely to acquire a territory of high quality [11,12], to breed [13,14,15] and to successfully rear numerous offspring relative to older ones [16,17,18,19].

Importantly, all these non-mutually exclusive hypotheses support the idea that young individuals should reproduce less well than older ones. Accordingly, most studies have so far focused on the ability of individuals to rear one or several offspring while neglecting the quality (*sensu* [20]: an axis of among-individual heterogeneity that is positively correlated with individual fitness) of the offspring produced (but see [15,21,22,23,24]). However, the quality of the offspring is certainly a crucial variable to focus on because the fitness of an individual obviously depends on the ability of its offspring to survive and reproduce [20]. In most bird species, post-fledging survival is usually much lower than adult survival, and only a small percentage of the offspring produced are able to recruit into the population [25,26,27]. This suggests that only high-quality offspring will survive and benefit to the fitness of their parents [28,29]. To better assess the influence of age on an individual’s performances, one should therefore not only focus on the ability of individuals to breed or produce young, but also on the ability of young to subsequently survive and reproduce [15,22,23,24]. However, such an approach is obviously very challenging in wild animal populations because this requires following a large number of offspring during their life, which is practically difficult to achieve except in a few specific study systems.

In that context, several proxies of individual quality have been used to assess the ability of young to survive and recruit into wild animal populations. For example, body size and body condition are usually considered as reliable proxies of developmental conditions [21,30]. Similarly, some physiological parameters, such as stress hormone levels, metabolism or immunity, are directly linked to developmental conditions (e.g., [31,32,33,34,35,36]). Despite their relevance when focusing on the phenotypic consequences of poor developmental conditions, all these variables could be labile and change rapidly once the offspring have left the natal environment (condition: [30]; metabolism: [33], 2008; stress hormones: [37]). Although several studies have shown that developmental conditions can translate into long-term effects on physiology, body condition, behavior [34,38,39,40], this is not always the case [36,41,42] and the impact of these long-term phenotypical effects on fitness are often complex and context- or sex-dependent (reviewed in [43]). In contrast, telomere length has recently been suggested as a promising molecular tool to assess the quality of the offspring because long telomeres have been directly linked to improved behavior and physiology [44,45,46,47]. Moreover, telomere length seems to be mainly determined during the developmental period (reviewed in [44]) and contrary to other physiological measurements, telomere length at the end of the developmental period has been convincingly linked to longevity and fitness in captive and wild animals [48,49].

Telomeres are terminal chromosomal complexes composed of highly repeated DNA sequences and proteins [50]. Telomere length is heritable but telomeres are also reduced during each cell division as a result of incomplete end-replication [50] and this telomere shortening is also accelerated by oxidative stress [51]. Importantly, telomere attrition appears to be accelerated by the occurrence of environmental or nutritional constraints [44,45,49,52,53,54,55], especially during the developmental period [44,52,56,57,58,59,60,61,62]. In altricial species, developmental conditions are obviously dependent on environmental conditions (food availability, predation risk, etc.) but they are also tightly connected with the quality of parental cares [63]. Therefore, offspring telomere length seems relevant to investigate the influence of environmental conditions and parental attributes, such as age, on offspring quality [44,45,64]. Although a few biomedical studies have examined this question in humans [65,66,67], we currently lack empirical studies that investigated the influence of parental age on offspring telomere length in wild animals across the fast-slow life-history continuum (but see [49,68,69,70,71]), particularly for species situated at the slow end of the continuum (i.e. long-lived species).

In this study, we tested whether parental age affects offspring telomere length in a long-lived bird species, the Black-browed Albatross (*Thalassarche melanophrys*). According to the selection, constraint and restraint hypotheses, we expect offspring quality to be positively correlated with parental age. We sampled fledglings from known-age parents in three consecutive years to monitor their telomere length. According to previous studies [8,72], we predict that young albatrosses should provide parental cares of low quality because of reduced parental/foraging skills (the constraint hypothesis), a reduced investment into reproduction (the restraint hypothesis), or because of a progressive disappearance of low-quality parents with advancing age (the selection hypothesis). Therefore, we predict that the offspring of young albatrosses should have shorter telomeres (prediction 1) and lower body condition (prediction 2) than those of older parents. If telomere length is a reliable proxy of developmental nutritional conditions [57,58,59,61], we also predicted that telomere length should be positively correlated with body condition (prediction 3).

## Material and methods

### Study species and determination of parental age

Our study focused on the Black-browed Albatross, a long-lived seabird species ([73]; maximum observed lifespan: 44 years) with a low fecundity (only one egg is laid per couple and per year, [73,74]). Sexual maturity is acquired on average at 11 years old and albatrosses then breed annually although a small proportion of birds skip breeding each year [75].

A 49-years old ongoing mark-recapture program (see [8,9,73,76]; Angelier et al., 2010; Nevoux et al., 2007; Pardo et al., 2013 for details on the monitoring protocol) has been conducted on approximately 200 breeding pairs at Cañon des Sourcils Noirs, Kerguelen archipelago, south-western Indian Ocean (50°S, 70°E). At this colony, all chicks have been banded every year since 1980. After fledging, juveniles spend a few years at sea and return to the colony to breed, allowing us to have access to many known-aged breeders. Each year, the bands of all breeding pairs are checked after egg-laying, allowing us to determine the age of both parents for several nests. For a large proportion of birds, the sex of breeders is known from previous studies that used molecular sexing techniques [77]. All nests are then checked twice during the breeding season to determine hatching and fledging success (respectively, in early January and late March).

### Morphometric measurements and feather sampling

This study was conducted during three consecutive breeding seasons (2011–2012, 2012–2013, 2013–2014). In late March, when the chicks were about to fledge, at approximately 3 months old, they were captured at their nest (n = 51; 15 in 2011–2012, 18 in 2012–2013, 18 in 2013–2014), banded and their beak length was measured with a caliper to the nearest 0.1mm in order to obtain a measure of body size [78]. All chicks were weighed to the nearest 50g using a spring balance. Body condition was then calculated by use of the scaled mass index (SMI) following [79]. Beak size was positively correlated with body mass (r = 0.419; F_1,49_ = 10.77; p = 0.002) and was therefore used to calculate the SMI using the following formula ${SMI}_{i}=M_{i}\times {\left(\frac{L_{0}}{L_{i}}\right)}^{b}$. The terms *M*_*i*_ and *L*_*i*_ respectively correspond to the body mass and the beak size of the offspring *i*. The term *L*_*0*_ is the arithmetic mean value of beak size for the whole study population (*L*_*0*_ = 111.06 mm, n = 51 individuals). The exponent *b* corresponds to the slope estimate of a standardized major axis regression of log-transformed body mass on log-transformed beak size (*b* = 1.904). Finally, a growing feather was plucked from the back of the chick to obtain DNA for telomere length determination and molecular sexing. The feather was stored at -20°C until subsequent molecular analyses.

### Molecular sexing and telomere analyses

For each feather, the bulb was extracted from the whole feather with a scalpel and was digested with proteinase K. DNA was then extracted from this sample by using the DNeasy blood and tissue kit (Qiagen). The sex of each chick was determined by polymerase chain reaction (PCR) amplification of the CHD gene following the standard procedures [77]. Telomere length was determined at the Centre d’Etudes Biologiques de Chizé by using the TeloTAGGG Telomere Length Assay (Roche, Mannheim, Germany) as previously described and with minor modifications [80]. DNA quality was checked by optical density spectrophotometry and gel electrophoresis. Preliminary tests were conducted to determine the optimal amount of DNA to be used to run this assay. Thus, 2 μg of DNA was digested with the restriction enzymes *Hinfl* and *Rsal* for 16 h at 37°C. Digested DNA samples were then separated with a pulse-field gel electrophoresis (Bio-Rad) on a 0.8% agarose gel. All samples were run in three gels and internal controls were used on each gel to measure inter-gel variations. The gels were run at 3.0 V/cm with an initial switch time of 0.5 s to a final switch time of 7 s for 14 h. The gel was then depurinated and denaturated in an alkaline solution. After neutralizing the gel, DNA was transferred onto a nitrocellulose membrane by Southern blot (Hybond H+, Amersham Life Science, Amersham UK). The membrane was incubated at 120°C for 20 min in order to fix the DNA. Finally, the DNA was hybridized with a digoxigenin-labeled probe specific for telomeric sequences, incubated with antidigoxigenin-specific antibody, and was visualized with a Chemidoc (Bio Rad). Telomere length was determined using ImageJ by analysing telomere smear densities. Mean telomere length was calculated in a window of 5–30 kb that includes the whole smear [81]. Inter CV was 1.76%.

### Statistical analysis

Because the influence of mean parental age on several demographic and physiological parameters may be non-linear [15,72], we first conducted preliminary analyses using generalized additive models (GAM, [82]) with smoothing splines to investigate the shape of the relationships for female and male offspring between our variables of interest (telomere length and SMI) and mean parental age, i.e. the mean of paternal and maternal ages (e.g. [83]). These preliminary analyses showed that both telomere length and SMI were linearly related to mean parental age (supplementary material Table A in S1 File). Therefore, we used linear models (LM) with a normal error distribution to test the relationships between (1) offspring telomere length and mean parental age, (2) offspring body condition and mean parental age, and (3) offspring body size (beak size) and mean parental age. For each model, mean parental age, the year of sampling, the sex of the offspring and their interactions were included as predictor variables. Maternal and paternal age could not be included simultaneously in the models as explanatory variables because they were highly correlated (r = 0.730, F_1,39_ = 35.26, p < 0.001). Moreover, the sex of parents could not be determined in 10 pairs limiting therefore our sample size (n = 41). Despite this limitation, we ran two model selections including either maternal age or paternal age as the age-related explanatory variable and compared their AICc in order to attempt to evaluate the relative importance of maternal and paternal age on offspring telomere length. The selected models had similar AICc values and we could not disentangle the influence of maternal and paternal age on telomere length. Therefore, we used mean parental age in the rest of the manuscript. The age of both parents could not be determined for two nests. Because paternal age and maternal age were highly correlated in the other pairs (n = 41), we used maternal or paternal age as a proxy of mean parental age for these two nests. Finally, we tested whether offspring telomere length was correlated with offspring body condition in males and females. We ran one regression per sex because body condition differed between female and male offspring, leading to potential multicollinearity problems [84]. Model selections were performed using a backwards stepwise approach starting from the most complex model and eliminating progressively explanatory variables or interactions with p ≥ 0.10. In addition, we also used the information-theoretic approach and the AICc to conduct model selections ([85]; see supplementary material Table B in S1 File). Results from both approaches were similar and we only present the results from the backwards stepwise approach in this article. We also describe how well the full models and the selected models fit the data for each model selection (r^2^, see supplementary material Table B in S1 File). All statistical analyses were performed with R.3.1.1 [86].

### Ethic statement

Licences and permissions were granted by the Ethic Committee of Institut Polaire Francais (IPEV) and by the Préfet of Terres australes et antarctiques francaises (TAAF) after advices from the Comité de l’Environnement Polaire (CEP).

## Results

### Influence of mean parental age on offspring telomere length

**Fig 1:**
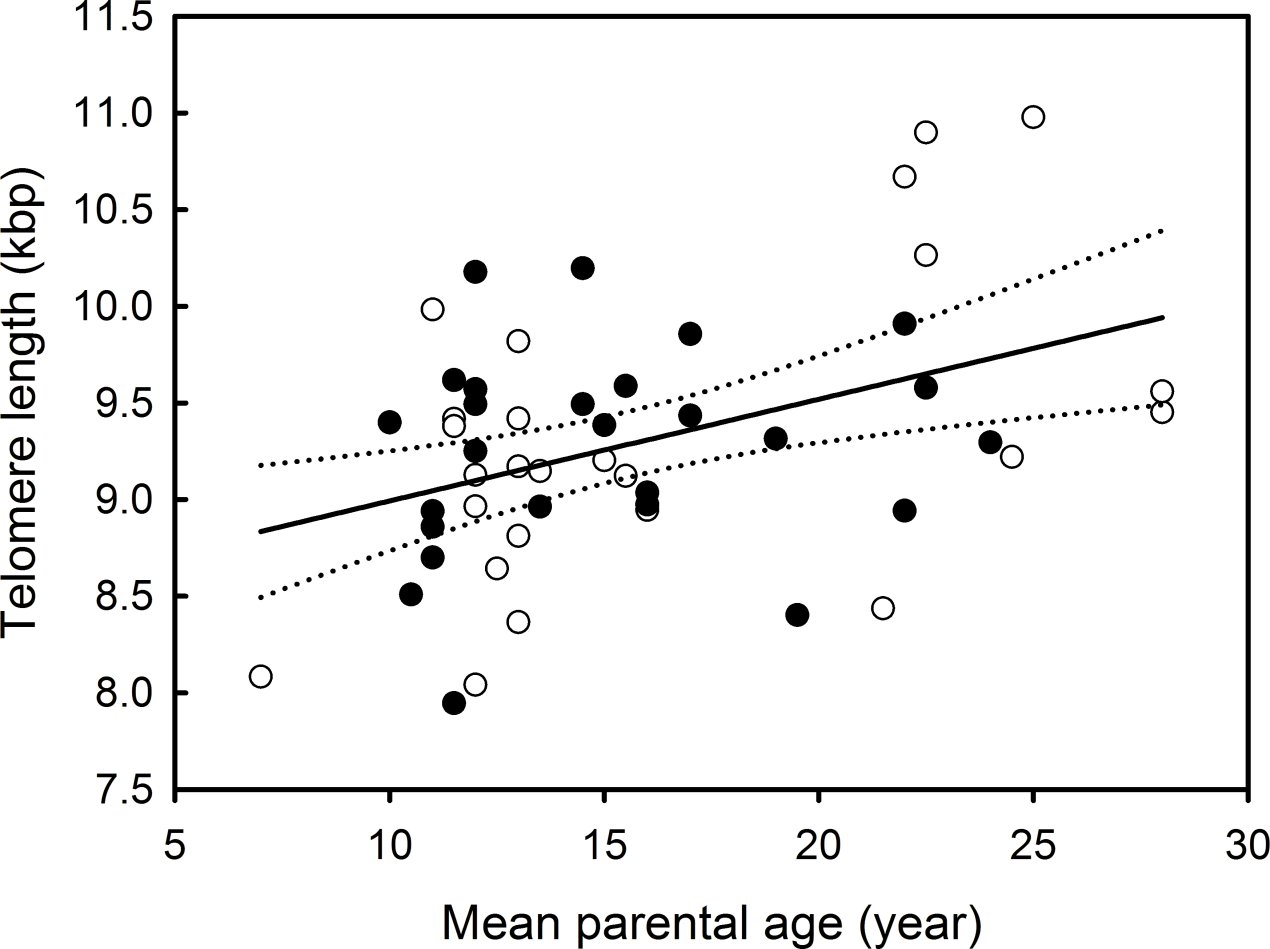
Relationship between offspring telomere length and mean parental age in Black-browed albatrosses. Filled and open circles respectively represent females and males. The solid line represents the relationship between offspring telomere length and parental age. The dotted lines represent the 95% confidence intervals for the relationship between offspring telomere length and mean parental age.

Mean parental age of sampled chicks was 15.706 ± 5.111 and varied from 7 to 28 years. Offspring telomere length was significantly and positively correlated with mean parental age (Table 1; Fig 1). However, offspring telomere length was not associated with the sex of the offspring, the year of sampling, or any interaction (Table 1).

**Table 1:** Model selection and LM to test the influence of mean parental age, sex of the offspring and the year of sampling and their interactions on offspring telomere length. The best model (in bold type) was selected by using a backwards stepwise approach starting from the most global model (models were simplified by eliminating independent variables with p ≥ 0·10). Parameter estimates are provided for the best model.

Variable of interest	Explanatory variables	df	F-value	p-value
Offspring telomere length	**Mean parental age**	**1.49**	**9.77**	**0.003**
	Sex of the offspring	1.46	0.002	0.962
	Year of sampling	2.47	2.36	0.106
	Mean parental age x sex	1.45	2.63	0.111
	Mean parental age x year of sampling	2.43	2.28	0.114
	Sex x year of sampling	2.41	2.44	0.1
Parameter	Estimate	Standard error	t-value	p-value
Intercept	8.46	0.278	30.4	<0.001
Mean parental age	0.053	0.017	3.12	0.003

### Influence of mean parental age on body condition and beak size

**Fig 2:**
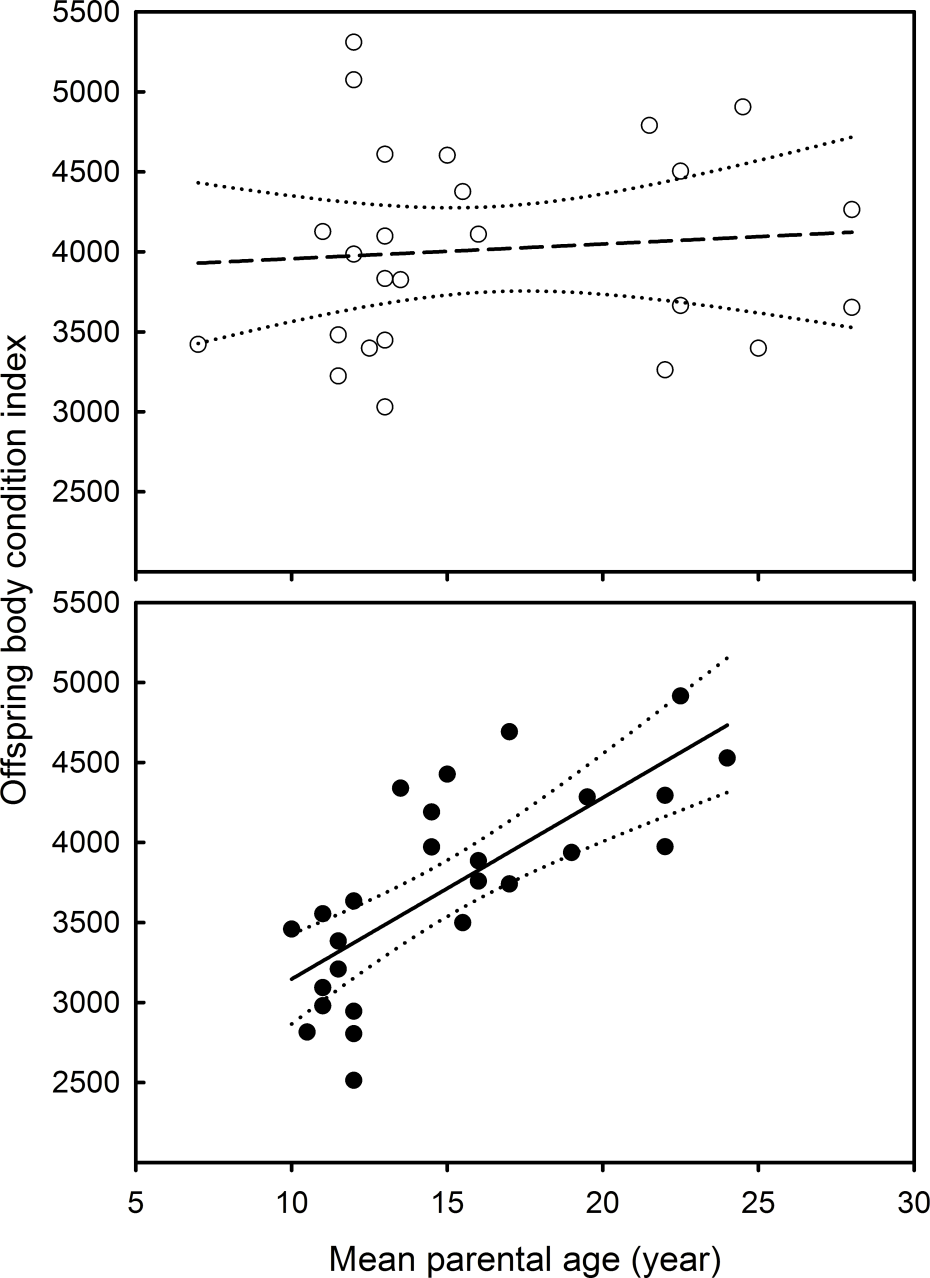
Relationship between offspring body condition and mean parental age in Black-browed albatrosses. Filled and open circles respectively represent females and males. The solid and dashed lines respectively represent the relationship between offspring body condition and parental age for females (significant) and males (non-significant). The dotted lines represent the 95% confidence intervals for these relationships.

Offspring body condition was associated with mean parental age, the sex of the offspring, and their interaction (Table 2). Specifically, offspring body condition increased with mean parental age in female offspring, but not in male offspring (Table 2; Fig 2). However, offspring telomere length was not affected by the year of sampling, or any other interaction (Table 2).

**Table 2:** Model selection and LM to test the influence of mean parental age, sex of the offspring and the year of sampling and their interactions on offspring body condition. The best model (in bold type) was selected by using a backwards stepwise approach starting from the most global model (models were simplified by eliminating independent variables with p ≥ 0·10). Parameter estimates are provided for the best model.

Variable of interest	Explanatory variables	df	F-value	p-value
Offspring body condition	**Mean parental age**	**1.47**	**18.8**	**<0.001**
	**Sex of the offspring**	**1.47**	**12.6**	**<0.001**
	Year of sampling	2.45	1.47	0.241
	**Mean parental age x sex**	**1.47**	**10.5**	**0.002**
	Mean parental age x year of sampling	2.41	0.038	0.963
	Sex x year of sampling	2.43	1.82	0.173
Parameter	Estimate	Standard error	t-value	p-value
Intercept	2013.3	408.4	4.93	<0.001
Mean parental age	113.3	26.1	4.34	<0.001
Sex of the offspring	1852.4	522.2	3.55	<0.001
Mean parental age x sex	-104.1	32.1	-3.24	0.002

Beak size was not affected by mean parental age, the year of sampling, or any interaction (Table 3). However, beak size differed between sexes with longer beak in male offspring relative to female ones (Table 3).

**Table 3:** Model selection and LM to test the influence of mean parental age, sex of the offspring and the year of sampling and their interactions on offspring beak size. The best model (in bold type) was selected by using a backwards stepwise approach starting from the most global model (models were simplified by eliminating independent variables with p ≥ 0·10). Parameter estimates are provided for the best models.

Variable of interest	Explanatory variables	df	F-value	p-value
Offspring beak size	Mean parental age	1.46	0.661	0.42
	**Sex of the offspring**	**1.49**	**4.72**	**0.035**
	Year of sampling	2.47	1.32	0.278
	Mean parental age x sex	1.41	0.719	0.401
	Mean parental age x year of sampling	2.42	0.763	0.472
	Sex x year of sampling	2.44	1.28	0.289
Parameter	Estimate	Standard error	t-value	p-value
Intercept	109.7	0.872	125.9	<0.001
Sex of the offspring	2.70	1.24	2.17	0.035

### Relationship between telomere length and body condition

Offspring telomere length was marginally and non-significantly associated with body condition in males (F_1,23_ = 3.032, p = 0.095). However, offspring telomere length was not associated with body condition in females (F_1,24_ = 0.875, p = 0.359).

## Discussion

In this study, we showed that mean parental age was positively correlated with offspring telomere length in a long-lived seabird species. We also found a clear connection between mean parental age and offspring body condition although this correlation was only apparent for female chicks. Because offspring condition and offspring telomere length have been linked with survival probability (condition: [29,87]; telomeres: [48,88]), our study therefore suggests that young parents are less likely to successfully rear high-quality offspring relative to older ones because of reduced parental skills (constraint hypothesis), reduced parental investment (restraint hypothesis) or/and the progressive disappearance of low-quality individuals with advancing age (the restraint hypothesis).

### Offspring telomere length and parental age

Consistent with our first prediction, we found that young Black-browed albatrosses raised chicks with shorter telomere relative to those raised by older ones. Accordingly, recent studies have also reported that offspring telomeres length is positively affected by parental age in two short-lived bird species, the great-reed warbler (*Acrocephalus arundinaceus*, [49]) and the white-throated dipper (*Cinclus cinclus*, [68]). However, this pattern was not found in other studies: recently, both a negative correlation and no correlation between these two variables were respectively obtained in the Alpine swift (*Tachymarptis melba*, [70]) and in the Soay sheep (*Ovis aries*, [71]). Therefore, the relationship between telomere length and parental age seems to be species- and/or context-dependent. Because short offspring telomeres have been linked to lower longevity and survival probability in several studies [48,62,88], our results suggest that younger parents produced offspring of lower quality.

How can we explain such an effect of parental age on offspring telomere length? First, heritability of telomere length could explain this pattern if individuals with short telomeres are more likely to reproduce at young ages. In wild vertebrates, telomere length is known to be somewhat heritable ([49,89,90,91,92]; but see [68]) but also to be related to longevity [48,88] and individual quality [93]. Therefore, a progressive disappearance of individuals with short telomeres may occur among a cohort as individuals become older [94]. Because telomere length is somewhat heritable [49,89,90,91,92], this could translate into a positive correlation between parental age and offspring telomere length and may therefore explain our results (“the selection hypothesis”). Supporting this possibility, survival probability is reduced at young ages in Black-browed albatrosses [9]. Therefore, low quality individuals (with short telomeres) may disappear from the population when they are still young and only high-quality individuals with longer telomeres may be found among the older albatrosses. Second, there is now strong evidence that constraining environmental conditions accelerate telomere attrition in developing individuals [44,45]. Specifically, nutritional constraints have been associated with short telomeres in the chicks of multiple species [57,58,62,95,96,97]. In addition, the occurrence of stressful events also leads to telomere attrition in developing vertebrates [59,60,62]. As a consequence, a lower parental effort and/or lower parental skills could be associated with shorter telomeres in offspring at the end of growth (“the constraint or the restraint hypotheses” although we cannot disentangle these two hypotheses). For example, younger Black-browed Albatross parents may provide low quality parental cares that are associated with developmental constraints/stress for the offspring [1], and therefore accelerated offspring telomere attrition. Supporting this hypothesis, young parents are often poor foragers [4,5,98], and therefore, they may only be able to provide scarce and/or low-quality food to their progeny during the demanding chick-rearing period [78,99]. Furthermore, they may also be less committed to protect their chick when stressors occur (e.g. inclement weather), leading potentially to elevated stress levels in their progeny, and therefore, to increased telomere attrition [46,53,56,59,62]. Accordingly, several studies have shown that younger albatrosses are more energetically constrained [99], have higher stress hormone levels (corticosterone, [72]), lower levels of parental care hormone (prolactin, [72]) and are overall less likely to reproduce successfully [8,9,100]. For practical reasons, we did not sample the parents in our study and we only sampled the chicks when they were about to fledge, aged 3 months, i.e. at the end of growth. Although this does not allow us to tease apart the relative importance of selection and telomere heritability, parental skills and parental effort (and therefore developmental conditions) in determining offspring telomere length, our study clearly shows that parental age has an important effect on offspring telomere length.

### Relative importance of maternal and paternal age in determining offspring telomere length

Paternal age and maternal age were strongly correlated in our study and maternal and paternal ages were very similar within couples. Therefore, it was difficult to estimate the relative importance of paternal age and maternal age in determining offspring telomere length. However, in recent studies, offspring telomere length was correlated to maternal age, but not to paternal age in the great-reed warbler and the white-throated dipper [49,68]. This suggests that maternal characteristics may play a more important role than paternal ones in determining offspring’s telomere length, at least in these short-lived species. In addition to heritability that does not seem to play a major role in explaining offspring telomere length in all species [68], several studies also found that parental age can affect offspring telomere length through an indirect effect of age on maternal [49,68,69,70] or paternal effort [69], and therefore, on offspring developmental conditions [57,58,62,95,96,97]. Contrary to the great-reed warbler and the white-throated dipper, parental effort is equally shared between female and male Black-browed albatrosses [78]. Therefore, both paternal and maternal age might impact offspring telomere length as it is the case in another seabird species, the European shag (*Phalacrocorax aristotelis*; [69]).

Although we sampled very old individuals in our study, we did not find any evidence that very old parents produce offspring with shorter telomeres. However, sample sizes of old individuals were low in our study for several reasons. Firstly, the reproductive success of Black-browed albatrosses decreases at very old ages [9,72] and onset of reproductive senescence only appears when parents reach at least 25–30 years old in this species [9]. The chicks of senescent birds often die before fledging and we have probably sampled very few offspring of senescent parents, resulting in a lack of statistical power to detect a potential effect of parental senescence on offspring telomere length.

### Parental investment in relation to the sex of the offspring

Importantly and consistent with our second prediction, we also found that young parents had chicks of lower body condition when compared with older parents. Surprisingly, this effect of parental age on offspring body condition was only apparent for female offspring and parental age was not correlated with the body condition of male offspring. Several hypotheses could potentially explain this pattern. Firstly, this sex-difference in the relationship between parental age and offspring condition could result from contrasted energy requirements between female and male chicks. If female offspring require more energy to grow than male ones, young parents may be able to efficiently raise male chicks, but not female chicks because of poorer parental skills. However, this hypothesis is very unlikely because females are slightly smaller than males ([101], our results on beak size), suggesting that raising a male offspring is slightly more energy-demanding than raising a female offspring (see [87] for the Wandering albatross *Diomedea exulans*). Second, this difference could result from a higher parental investment towards male offspring relative to female ones. Thus, young individuals may increase their parental effort when raising a male chick and this might allow them to compensate for their potential poorer parental skills [87]. Finally, this pattern could also result from a covariation between individual quality and offspring sex ratio: in Wandering albatrosses, high-quality parents produce more sons than daughters [102]. Because of better foraging skills, young high-quality parents could more efficiently sustain the energetic needs of their offspring (mainly males) while young low-quality parents may not be able to do so with their offspring (mainly females).

When raised by young parents, female chicks are in poorer condition than male chicks but they also have short telomeres, confirming therefore that their developmental conditions were probably particularly constraining. On the other hand, body condition of male chicks is not influenced by parental age and the male offspring raised by young parents have a similar body condition to those raised by older parents. Although this suggests that young parents may be able to sustain the energetic needs of their sons, this may not hold totally true when analyzing the telomere length of male offspring. When compared to older parents, young parents raise daughters, but also sons, with shorter telomeres. This suggests that male offspring may also suffer from constraining developmental conditions despite their relatively good body condition at fledging. How can we explain these *a priori* contradictory results?

We can raise the following hypothesis. When raising a son, young parents seem to be able to sustain the growth and energetic needs of their offspring. However, young pairs of parents are known to be less synchronized than older ones, and this probably results in irregular and erratic chick provisioning, as demonstrated in a closely-related species [87]. Overall, young parents may increase their parental investment when raising a son, explaining therefore that parental age does not have an impact on male offspring body condition. When raised by inexperienced and poorly synchronized young parents, male chicks may however undergo temporary periods of nutritional deficits that are then compensated by increased parental investment, and thus, periods of intense feeding. Such temporary nutritional constraints are known to result in irregular growth [38], and more importantly, accelerated telomere attrition [57,58,96,97,103,104] although it does not necessarily have a detrimental impact on fledging size and body condition [105]. This hypothesis needs now to be properly tested to fully understand why our results differ depending on the sex of the offspring.

### Offspring telomere length and body condition

Contrary to our third prediction, we did not find any correlation between offspring telomere length and body condition. This result is quite surprising because both telomere length and body condition were positively related to parental age (at least in females). This may result from a large inter-individual variance in both telomere length and body condition that might have blurred the relationship between telomere length and body condition in our study. Offspring telomere length is certainly affected by developmental conditions, but also by initial telomere length through heritability [49,89,90,91,92]. In addition, body condition is highly labile in old albatross chicks because parent albatrosses feed their large chicks quite irregularly [78]. Therefore, our measure of body condition may be quite variable because it certainly depends on the time of the last chick-feeding event, which was not measured in our study. This inter-individual variance in both telomere length and body condition might explain why we did not find any significant relationship between offspring body condition and telomere length. Future studies should carefully examine the functional relationship that link offspring body condition, parental feeding, telomere heritability and offspring telomere dynamics.

In conclusion, we demonstrated in this study that parental age has a strong influence on offspring body condition and offspring telomere length in a long-lived bird species. Because both body condition and telomere length depend on developmental conditions, our study suggests that older individuals might be able to provide better parental care to their offspring, probably because of better foraging skills and/or higher parental investment (the constraint/restraint hypotheses). In addition, this apparent improvement of offspring condition with advancing parental age may also result from the disappearance of low-quality individuals at young ages (the selection hypothesis). Importantly, we found a detrimental influence of young parental age on offspring telomere length in both sons and daughters. However, the detrimental influence of young parental age on offspring body condition was apparent in daughters only. Although male offspring are in good body condition at fledging when raised by young parents, they still have short telomeres. Finally, offspring body condition and telomere length were not correlated, and our study therefore highlights the need of looking at several complementary proxies of fitness to fully understand the impact of parental or environmental characteristics on offspring fitness.

## Supporting information

S1 File Additional analyses. (PDF)

S2 File Data used for all analyses. (PDF)
