# Supplementary material for: Young parents produce offspring with short telomeres: A study in a long-lived bird, the Black-browed Albatross (Thalassarche melanophrys)
Source: PLoS One. 2018 Mar 21;13(3):e0193526. doi: 10.1371/journal.pone.0193526 (PMC5862442; doi:10.1371/journal.pone.0193526)
Supplement: S1 File — (PDF) [file pone.0193526.s001.pdf]

S1 file

Table A: Generalized additive models (GAM) with smoothing splines were used to investigate the shape of the relationships for female and male offspring between our variables of interest (telomere length and SMI) and parental age. These preliminary analyses showed that both telomere length and SMI were linearly related to parental age.

Variable of interest	Chick's sex	Predictor variable	df	F-value	p-value	%Devaince explained
Offspring telomere length	Female	s(Age)	1	0.684	0.416	2.77
	Male	s(Age)	1	8.71	0.007	27.5
Offspring body condition	Female	s(Age)	1.58	18.5	< 0.001	58.7
	Male	s(Age)	1	0.173	0.681	0.746

Table B: LM models selection table based on the Akaike Information Criterion (AICc) to determine the best models when investigating the influence of mean parental age, chick's sex and year of sampling on offspring telomere length. Presence (+) or absence (-) for qualitative effects, AICc computations and relative variable importance (Weight) are indicated for each model. Best models are shown in bold. The r^2 of the full model and the best-fitting model were respectively 0.295 and 0.149; 0.290 and 0.282; 0.069 and 0.069 for offspring telomere length, offspring body condition and offspring beak size.

Offspring telomere length

Model	Mean parental age	Year of sampling	Chick's sex	Mean parental age x year of sampling	Mean parental age x Chick's sex	Chick's sex x year of sampling	AICc	deltaAICc	Weight
Model 1	+	+					98.6	0.000	0.241
**Model 2**	+						**98.7**	**0.050**	**0.235**
Model 3	+	+		+			101.0	2.36	0.074
Model 4	+	+	+		+		101.0	2.37	0.074
Model 5	+		+				101.0	2.41	0.072
Model 6	+	+	+				101.2	2.57	0.067
Model 7	+		+		+		101.6	2.98	0.054
Model 8	+	+	+	+	+		101.6	3.01	0.053
Model 9	+	+	+	+	+	+	102.3	3.65	0.039

Offspring body condition

Model	Mean parental age	Year of sampling	Chick's sex	Mean parental age x year of sampling	Mean parental age x Chick's sex	Chick's sex x year of sampling	AICc	deltaAICc	Weight
**Model 1**	+		+		+		**794.8**	**0.000**	**0.606**
Model 2	+	+	+		+		796.9	2.05	0.218
Model 3	+	+	+		+	+	798.5	3.68	0.096
Model 4	+	+					802.2	7.35	0.015
Model 5	+						802.3	7.5	0.014
Model 6	+	+	+	+	+		802.5	7.7	0.013
Model 7	+		+				802.7	7.82	0.012
Model 8	+	+	+				803.3	8.43	0.009
Model 9	+	+	+	+	+	+	804.8	9.97	0.004

Offspring beak size

Model	Mean parental age	Year of sampling	Chick's sex	Mean parental age x year of sampling	Mean parental age x Chick's sex	Chick's sex x year of sampling	AICc	deltaAICc	Weight
**Model 1**			+				**301.4**	**0.000**	**0.343**
Model 2	+		+				303.1	1.74	0.144
Model 3		+	+				303.4	2.04	0.123
Model 4							303.8	2.43	0.102
Model 5	+						305.0	3.67	0.055
Model 6		+					305.2	3.87	0.049
Model 7	+	+	+				305.3	3.89	0.049
Model 8	+		+		+		305.5	4.18	0.042
Model 9		+	+			+	306.5	5.17	0.026
Model 10	+	+	+	+	+	+	314.5	13.17	0.000

19
