# Supplementary material for: Young parents produce offspring with short telomeres: A study in a long-lived bird, the Black-browed Albatross (Thalassarche melanophrys)
Source: PLoS One. 2018 Mar 21;13(3):e0193526. doi: 10.1371/journal.pone.0193526 (PMC5862442; doi:10.1371/journal.pone.0193526)
Supplement: S2 File — (PDF) [file pone.0193526.s002.pdf]

Chick telomere length	Cohort	Parental mean age	Chick sex	Chick SMI
9,57	2011	12	F	2513,54
8,04	2011	12	M	5075,15
9,25	2011	12	F	2944,71
8,51	2011	10,5	F	2815,48
8,95	2011	16	M	4110,43
7,95	2011	11,5	F	3208,09
8,70	2011	11	F	2979,19
8,44	2011	21,5	M	4789,40
9,43	2011	17	F	3741,07
10,18	2011	12	F	2804,67
8,64	2011	12,5	M	3397,57
9,30	2011	24	F	4527,96
10,90	2011	22,5	M	3664,47
10,20	2011	14,5	F	3971,91
10,98	2011	25	M	3397,57
9,42	2013	11,5	M	3223,31
9,40	2013	10	F	3458,34
9,15	2013	13,5	M	3825,28
9,03	2012	16	F	3758,71
9,58	2012	22,5	F	4914,88
9,17	2012	13	M	3029,58
9,86	2012	17	F	4691,72
9,38	2012	11,5	M	3480,74
8,96	2012	13,5	F	4339,31
9,82	2012	13	M	3446,84
9,13	2012	12	M	3985,69
9,12	2012	15,5	M	4375,25
9,59	2012	15,5	F	3498,93
8,94	2012	22	F	3972,55
8,94	2012	11	F	3092,67
10,67	2012	22	M	3261,37
9,62	2012	11,5	F	3384,23
9,98	2012	11	M	4125,80
9,42	2012	13	M	4098,26
9,49	2012	14,5	F	4190,08
10,26	2012	22,5	M	4504,48
8,86	2013	11	F	3554,08
9,45	2013	28	M	4264,34
8,97	2013	16	F	3886,11
9,39	2013	15	F	4426,01
9,56	2013	28	M	3653,02
8,40	2013	19,5	F	4284,10
9,49	2013	12	F	3634,30
8,81	2013	13	M	4609,57
9,22	2013	24,5	M	4905,26
8,96	2013	12	M	5310,33
9,32	2013	19	F	3936,89
9,91	2013	22	F	4293,95
8,08	2013	7	M	3421,67

8,37	2013	13	M	3832,67
9,20	2013	15	M	4603,16
